# Graph-based molecular Pareto optimisation†

**DOI:** 10.1039/d2sc00821a

**Published:** 2022-06-02

**Authors:** Jonas Verhellen

**Affiliations:** Centre for Integrative Neuroplasticity, University of Oslo N-0316 Oslo Norway jverhell@gmail.com

## Abstract

Computer-assisted design of small molecules has experienced a resurgence in academic and industrial interest due to the widespread use of data-driven techniques such as deep generative models. While the ability to generate molecules that fulfil required chemical properties is encouraging, the use of deep learning models requires significant, if not prohibitive, amounts of data and computational power. At the same time, open-sourcing of more traditional techniques such as graph-based genetic algorithms for molecular optimisation [Jensen, *Chem. Sci.*, 2019, **12**, 3567–3572] has shown that simple and training-free algorithms can be efficient and robust alternatives. Further research alleviated the common genetic algorithm issue of evolutionary stagnation by enforcing molecular diversity during optimisation [Van den Abeele, *Chem. Sci.*, 2020, **42**, 11485–11491]. The crucial lesson distilled from the simultaneous development of deep generative models and advanced genetic algorithms has been the importance of chemical space exploration [Aspuru-Guzik, *Chem. Sci.*, 2021, **12**, 7079–7090]. For single-objective optimisation problems, chemical space exploration had to be discovered as a useable resource but in multi-objective optimisation problems, an exploration of trade-offs between conflicting objectives is inherently present. In this paper we provide state-of-the-art and open-source implementations of two generations of graph-based non-dominated sorting genetic algorithms (NSGA-II, NSGA-III) for molecular multi-objective optimisation. We provide the results of a series of benchmarks for the inverse design of small molecule drugs for both the NSGA-II and NSGA-III algorithms. In addition, we introduce the dominated hypervolume and extended fingerprint based internal similarity as novel metrics for these benchmarks. By design, NSGA-II, and NSGA-III outperform a single optimisation method baseline in terms of dominated hypervolume, but remarkably our results show they do so without relying on a greater internal chemical diversity.

## Abstract 2

Chemical diversity in Pareto optimization is sufficiently ensured by the structure of the algorithms, and outperforms an explicit quality-diversity approach.

## Graphical abstract

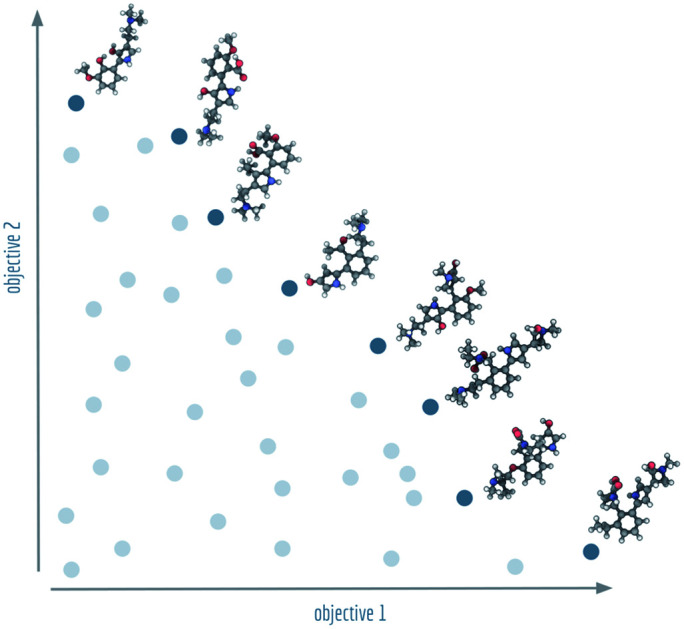

## 1 Introduction

Machine learning has recently assumed a prominent role^1^ in chemistry: predicting ADMET properties,^2^ supporting molecular dynamics simulations,^3^ and assisting in the design of small molecules without reverting to explicit rules or expert knowledge.^4–12^ However, training-free optimisation algorithms that comprehensively traverse and explore chemical space have been shown to be more efficient^13,14^ than their machine learning counterparts in discovering high-performing *de novo* molecules. Sometimes this search in chemical space reduces to an optimisation for a single property like melting point^15^ or protein binding affinity,^16^ but often there are additional requirements that make it necessary to optimise for additional properties such as low toxicity,^17^ high synthesizability^18^ or off-target activity. In the case that multi-objective optimisation is necessary, a trade-off between different (and possibly competing) optimisation objectives has to be defined.

**Fig. 1:**
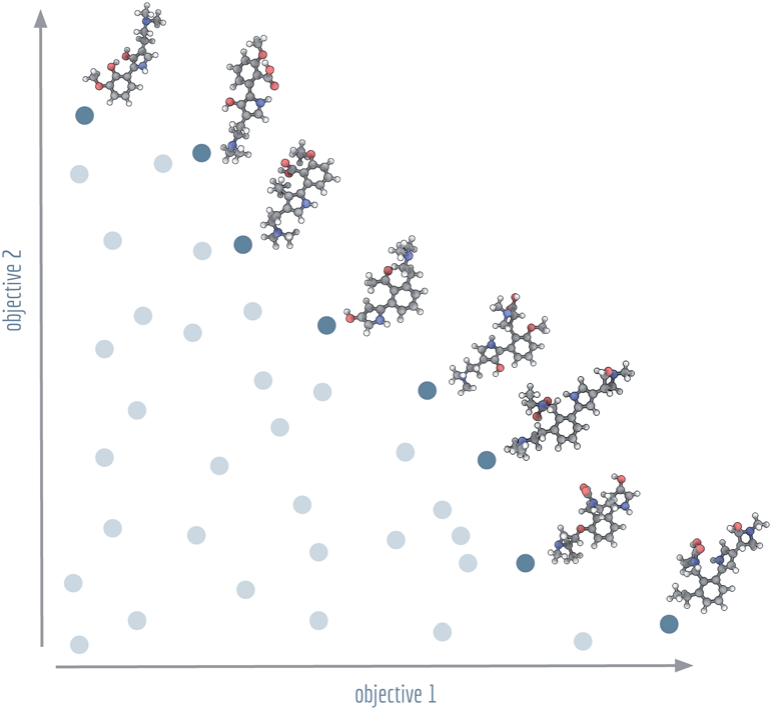
Visualistion of a Pareto front (dark blue) and dominated solutions (light blue). Example molecules shown at the Pareto front were generated by NSGA-II for Tanimoto similarities with regard to lysergic acid diethylamide (objective 1) and psilocybin (objective 2).

In current molecular generative model benchmarks,^13^ typically either the arithmetic mean or the geometric mean of the objective is chosen as a stand-in aggregate fitness function. To give relative importance to the different objectives, domain experts can assign weights to them or combine appropriate modifying functions to obtain a single, fine-tuned objective function. However, many fields of science and engineering make use of an alternative approach to multi-objective optimisation by searching for a set of so-called Pareto optimal solutions.^19^ All solutions in a Pareto optimal set are characterised by the fact that there are no other individual solutions that have a higher (or equal) fitness in all objective functions. Together, the set of Pareto optimal solutions form an optimal envelope in objective space known as the Pareto front, see Fig. 1.

The Pareto front provides a family of solutions, all equivalent in principle, aiding domain experts to make choices when trade-offs between objectives are not known beforehand. Over the past two decades, a set of algorithms known as the non-dominated sorting genetic algorithms^20^ (NSGA) has been developed for finding Pareto fronts. In a complex process, such as drug design, having access to a technique complementary to single objective optimisation, can yield deeper insights and improve efficiency. Therefore, in this paper, we provide the community with state-of-the-art and open-source implementations of the NSGA-II and NSGA-III algorithms^21–23^ based on a popular graph-based genetic algorithm^24^ (GB-GA) for molecular optimisation.

A newer generation of NSGA algorithm, NSGA-III, which uses a more complex means of ensuring coverage of the entire Pareto front, was originally reported to be an improvement over NSGA-II. However, later analyses^25,26^ have shown that for a wide range of computational experiments NSGA-III does not consistently outperform NSGA-II in every use-case. Therefor we compare the performance of NSGA-III and NSGA-II on a set of small molecule multi-objective optimisation benchmarks, making use of the dominated hypervolume as a novel measure of the effectiveness in these type of problems. As a baseline, we make use of a state-of-the-art single-objective optimisation algorithm that employs the geometric mean as a surrogate aggregate fitness function. Whereas proprietary applications of NSGA-II to molecular design have been reported,^27,28^ there is a lack of open-source implementations of both NSGA-II and NSGA-III for the inverse design of small molecules. We anticipate that our results and the availability of the code will encourage the development of more powerful Pareto optimisation algorithms for chemistry as well as their widespread adoption in computer-assisted chemical design.

## 2 Algorithmic methodology

NSGA-II and NSGA-III are genetic algorithms tailored to finding Pareto fronts. In this section, we introduce the fundamentals of genetic algorithms in the context of small molecule design and discuss the importance of balancing quality with diversity. We then describe the general framework of non-dominated sorting genetic algorithms and elaborate upon the NSGA-II and NSGA-III algorithms and their differences. In the remainder of the section, we discuss technical aspects such as structural alert based chemical filters, memoisation, the construction of reference directions (only used in NSGA-III), positional analogue scanning, and parallelism.

### 2.1 Genetic algorithms

A genetic algorithm is, as the name suggests, a heuristic search method^29^ inspired by the process of natural evolution. Genetic algorithms^30,31^ can achieve highly effective single-objective optimisation by consistently and incrementally improving a selection of trial solutions. The current set of the solutions used by the algorithm is known as the (evolutionary) population. In each iteration of the algorithm – known as a generation – novel solutions are generated by stochastically changing or combining the current solutions. In the genetic algorithm community, these two operations for generating new solutions are known as *mutations* and *crossovers*, respectively. At the end of each generation, the population is reduced to its original size by selecting only the highest performing molecules for survival. Eventually, the selection pressure in this procedure forces the population of solutions towards an optimum.

**Fig. 2:**
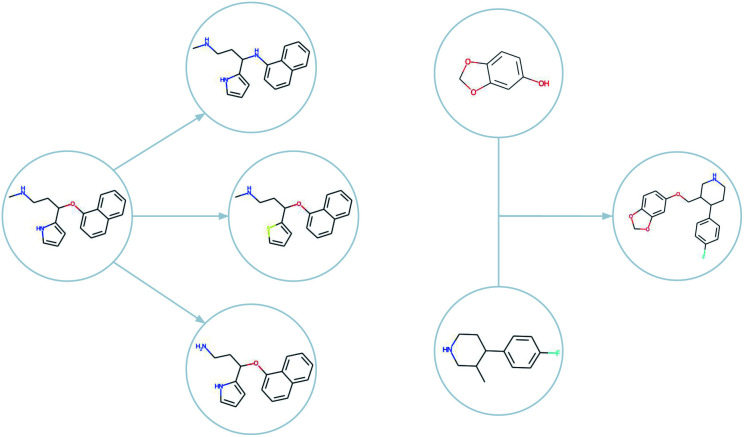
Examples of mutations (left) and a crossover (right) as generated by GB-EPI. Note that minor changes to chemical structure can be used to efficiently achieve optimisation even for challenging objectives.

For small molecule optimisation, these ideas can be implemented by representing solutions (*i.e.* molecules) by either their molecular graphs, or by text representation such as the simplified molecular-input line-entry system^32^ (SMILES) or self-referencing embedded strings^33^ (SELFIES). The graph representation has been used in the graph-based genetic algorithm (GB-GA) which was shown to outperform machine learning approaches.^24^ In Fig. 2, we show examples of mutations and crossovers on molecular graphs. To rule out graphs that represent impossible chemical configurations, only those that can be correctly translated to and from SMILES are retained. The initial population of candidate molecules is typically obtained from public databases like ZINC^34^ or ChEMBL.^35^

### 2.2 Quality-diversity algorithms

Unfortunately, genetic algorithms are known to be vulnerable to evolutionary stagnation when encountering low-performing valleys or local optima.^36^ Enforcing diversity^37^ in the population of molecules a genetic algorithm uses can alleviate these issues. Quality-diversity algorithms,^38^ such as the graph-based elite patch illumination algorithm^39^ (GB-EPI), obtain this diversity by splitting the population into niches based on their physicochemical properties. In each generation, the best performing molecule in each of the individual niches is retained, rather than selecting the highest-scoring solutions regardless of their diversity.

Alternatively, the superfast traversal, optimisation, novelty, exploration and discovery algorithm^40^ (STONED) leverages molecular diversity through the use of SELFIES. In contrast to the more traditionally used SMILES, SELFIES can be mutated arbitrarily at any position in the string to produce new strings that represent valid molecular structures. The STONED algorithm uses this property of SELFIES to preserve diversity in its population. By varying the position of modification within the string, the algorithm balances exploration and exploitation to avoid stagnation in low-performing valleys or local optima.

### 2.3 Non-dominated sorting genetic algorithms

In contrast to single-objective optimisation problems, in which diversity had to be discovered as a useable resource, diversity is inherently present in multi-objective optimisation problems. The presence of diversity is most obvious when considering a Pareto front, in which solutions to multi-objective optimisation problems must involve trade-offs to satisfy the conflicting demands of different objective functions. Several algorithms with different properties and varying levels of complexity have been proposed for finding Pareto optimal fronts. The main class of algorithms used for this task are the non-dominated sorting genetic algorithms, NSGA-II and NSGA-III.

**Fig. 3:**
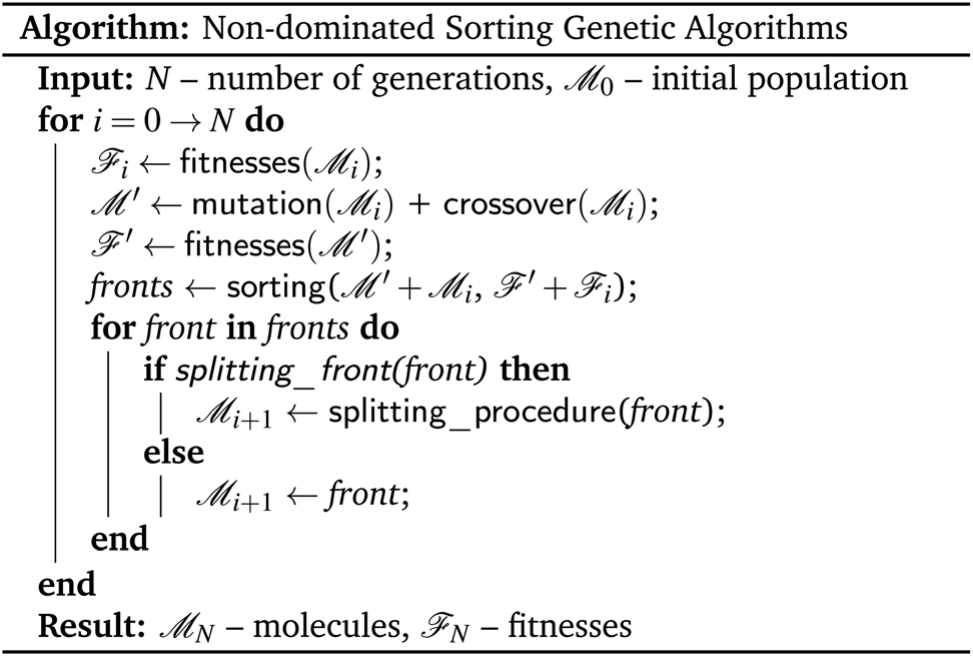
Pseudocode description of a generic non-dominated sorting genetic algorithm adapted to the setting of molecular optimisation.

**Fig. 4:**
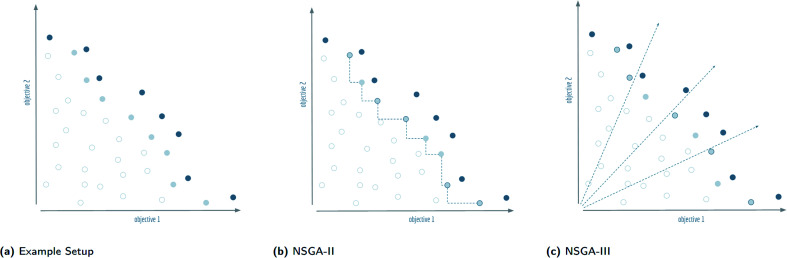
Visualisation of the splitting front procedure of non-dominated sorting genetic algorithms: (a) the Pareto dominant front is shown in dark blue, the splitting front is light blue, and the remaining solutions are white. For this example, the second front is chosen as the splitting front, and it is assumed that five more solutions need to be picked to complete the population. These solutions will be indicated with a dark blue circumference. (b) The selection procedure of NSGA-II calculates a distance in objective space to the nearest neighbours in the front. The outermost solutions are picked by default, the remaining solutions are chosen according to the furthest distance from neighbours. (c) The selection procedure of NSGA-III calculates the orthogonal distance to predefined reference directions in objective space and selects the closest solution for each axis. Note that the two objective axes are also used as reference directions so that the outermost solutions are picked by default.

Non-dominated sorting genetic algorithms^20^ are, in essence, genetic algorithms that evaluate and select on the Pareto dominating status of each solution in the evolutionary population as shown in Fig. 3. Instead of selecting molecules based on a fitness function, these algorithms sort all solutions into a series of fronts, see Fig. 4(a), each front dominated by the previous fronts. The first front (dark blue) is the set of completely non-dominated individuals in the current population, the second front (light blue) is the set of individuals dominated only by the individuals in the first front, and so on for all other fronts formed by the remaining individuals in the population (white). The algorithm accepts the fronts, with all of its individuals, into the evolutionary population in ascending order, until the maximum size of the evolutionary population has been reached.

The final front accepted by a non-dominated sorting genetic algorithm might, and often will, contain more individuals than can be added to the surviving evolutionary population without exceeding its size limit. This set of individuals is known in the multi-objective optimisation community as the *splitting front*.^20^ Because there is no difference between the individuals in the splitting front in terms of Pareto dominance, further criteria are used to select which individuals are retained and which are discarded. In the splitting front selection procedure for non-dominated sorting genetic algorithms, this criteria is typically a measure of diversity. The NSGA-II and NSGA-III algorithms both rely on a diversity criteria, but differ significantly in how they enforce this diversity, see Fig. 4(b) and (c).

### 2.4 NSGA-II

NSGA-II^21^ makes use of a *crowding distance* to differentiate within the splitting front. The crowding distance is calculated for each individual, and indicates how closely the individual is surrounded by the other members of the splitting front. For NSGA-II, the crowding distance used is the Manhattan distance^41^ in objective space. A larger crowding distance indicates a less crowded individual. Within a splitting front, NSGA-II orders all individuals by their crowding distances, and subsequently accepts the molecules with the largest crowding distance into the evolutionary population until the maximum size is reached. The outer solutions in the splitting front are assigned an infinite crowding distance to ensure that they are retained in each generation.

### 2.5 NSGA-III

In contrast to NSGA-II, the NSGA-III algorithm,^22,23^ uses reference directions^42,43^ instead of a crowding distance to enforce diversity in the selection of solutions within the splitting front. Reference directions are determined by a predefined set of points on the unit simplex in fitness space. Each reference direction is defined as a ray originating from the origin and passing through exactly one of these points. NSGA-III assigns a reference direction to each solution in the population based on the nearest perpendicular distance (in normalised fitness space) to the corresponding direction. In the splitting front selection procedure, the NSGA-III algorithm prioritises reference directions that are underrepresented in the current surviving evolutionary population.

If a reference direction does not have any solution assigned to it after reaching the splitting front, then the molecule in the splitting front with the smallest perpendicular distance to this direction is selected for survival. If all underrepresented reference directions have been assigned one surviving solution, and the maximum size of the surviving population has not been reached, the remaining solutions are selected by a stochastic procedure. Note that NSGA-III selects the solutions in the fronts before the splitting front in its entirety, like in NSGA-II. However, contrary to NSGA-II's crowding distance which is calculated within the splitting front, the reference directions used in NSGA-III take into account the diversity of the entire surviving population.

### 2.6 Reference directions

The reference directions determine the diversity in the selection of solutions from the splitting front, so these directions are typically chosen to be well distributed over the unit simplex. Traditionally the reference direction generation method of Das and Dennis has been used for NSGA-III. Unfortunately, due to the highly structured (combinatorial) nature of the Das–Dennis reference direction generating procedure,^42^ the method cannot produce an arbitrary number of directions. In addition, it has been shown that most of the reference directions generated by the Das–Dennis method cross through the boundaries of the unit simplex rather than the interior,^44^ inducing a bias in the selection of solutions from the splitting front.

To alleviate the issues of the Das–Dennis method, an energy-based approach has recently been proposed^43^ in the multi-objective optimisation literature. Inspired by methods in physics, a generalisation of the potential energy called the *Riesz s-energy*^45^ is calculated for a given number of reference points on the unit simplex. The Riesz *s*-energy *U*_*s*_ is defined between two points *p*_1_, *p*_2_ in *s*-dimensional Euclidean space as,1
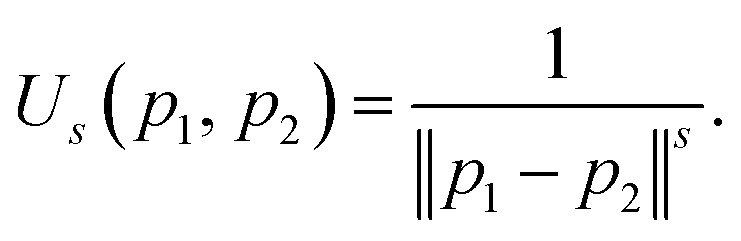


The location of the points along of the unit simplex are then optimised to minimise the combined Riesz *s*-energy of all the reference points. This allows for the construction of an arbitrary number of well-spaced reference directions. The results in this paper were obtained using the Riesz *s*-energy method to generate the reference directions for NSGA-III, with *s* equal to the square root of the number of objective functions as suggested in the original paper.^43^

### 2.7 Shared technical properties

We follow the example of GB-EPI^39^ and include a series of minor but important technical features to our NSGA-II and NSGA-III implementations, focused on improved chemical optimisation or higher relevance and better quality of the generated molecules. For instance, our NSGA-II and NSGA-III implementations make use of decoupled crossovers and mutations. As shown in GB-EPI, early on in an evolutionary algorithm, crossovers support the efficient exploration of chemical space, while later on local mutations are beneficial in improving the nearly-converged solutions. Therefore it is beneficial to apply both operators separately rather than in sequence.

Similarly, we follow the example of GB-EPI to apply the computational equivalent of *in vitro* positional analogue scanning^46^ by repurposing the mutation operator to systematically return not just a single mutation of a molecule, but all of its positional analogues. To offset the computational overhead introduced by positional analogue scanning and to improve efficiency in general, we store a record of obtained fitness calculations. This approach is known as memoisation^47^ and ensures that an algorithm does not unnecessarily repeat calculations. To further reduce clock time, we also implemented concurrency for the objective function evaluations and remove undesirable compounds based on structural ADMET filters^48–50^ before they enter the evaluation step of the algorithm.

## 3 Benchmarks

To the test the potency of our open-source implementations of NSGA-II and NSGA-III for multi-objective optimisation in drug design, we extend the use of tasks devised in the GuacaMol benchmarking suite^13^ by the bioinformatics company BenevolentAI. From the suite we selected multi-parameter optimisation (MPO) tasks with three or more objectives that aim to fine-tune the structural or physicochemical properties of five FDA-approved drugs: cobimetinib (a mitogen-activated kinase inhibitor), fexofenadine (a second-generation antihistamine), osimertinib (a tyrosine kinase inhibitor), perindopril (a long acting ACE inhibitor), and ranolazine (an anti-anginal drug). We search for a set of molecules that span the entirety of the Pareto front instead of trying to optimise a single value like the geometric mean.

The objectives in these benchmarks, as shown in Table 1, are either similarity metrics that measure the distance to the corresponding drug molecule, or specific properties such as the amount of rotatable bonds in a molecule, the topological polar surface area^52^ (TPSA) or the lipophilicity partition coefficient^53^ (log(*P*)). The similarity metrics are calculated using the Tanimoto similarity,^54,55^ of the fingerprints of the target and the generated candidate molecule. The fingerprints used here are either extended-connectivity fingerprints^56,57^ (ECFP/FCFP) which encode molecular structures in terms of concentric atomic neighbourhoods, or atom-pair fingerprints^58^ (AP) which encode molecules based on their atom pairs and their bond distance. The main advantage of fingerprint-based similarities compared to more involved similarity measures is that they can be rapidly calculated and inherently represent the presence or absence of molecular substructures or atom pairs.

**Table 1:** Overview of the multi-objective optimisation benchmarks used in this paper, the first five benchmarks are adapted from the Guacamol suite while the latter two benchmarks were constructed to emulate the demands of poly-pharmacology projects. The upper row of each task represents the values calculated for each objective. The lower rows show the modifiers applied to each of these values. The fingerprints used to calculate the similarities are denoted as arguments of the Tanimoto function, the parameters used for the modifiers are displayed as arguments of the corresponding functions. For the poly-pharmacology benchmarks, the genes targeted for activity are indicated. The CNS function calculates the central nervous system desirability score (high blood–brain-barrier permeability and low toxicity potential) as proposed by Pfizer^51^

Task\objective	I	II	III	IV	V
**Cobimetinib**					
	Tanimoto(FCFP4)	Tanimoto(ECFP6)	Rotatable bonds	Aromatic rings	CNS(0.5)
	Clipped(0.7)	MinGaussian(0.75, 0.1)	MinGaussian(3, 1)	MaxGaussian(3, 1)	—

**Fexofenadine**					
	Tanimoto(AP)	TPSA	log(*P*)	—	—
	Clipped(0.8)	MaxGaussian(90, 10)	MinGaussian(4, 1)	—	—

**Osimertinib**					
	Tanimoto(FCFP4)	Tanimoto(ECFP6)	TPSA	log(*P*)	—
	Clipped(0.8)	MinGaussian(0.85, 0.1)	MaxGaussian(95, 20)	MinGaussian(1, 1)	—

**Pioglitazone**					
	Tanimoto(ECFP4)	Molecular weight	Rotatable bonds	—	—
	Gaussian(0, 0.1)	Gaussian(356, 10)	Gaussian(2, 0.5)	—	—

**Ranolazine**					
	Tanimoto(AP)	log(*P*)	TPSA	Fluorine count	—
	Clipped(0.7)	MaxGaussian(7, 1)	MaxGaussian(95, 20)	Gaussian(1, 1)	—

**DAP kinases**					
	hERG	SCN2A	DAPk1	DRP1	ZIPk
	Gaussian(0, 0.1)	Gaussian(0, 0.1)	Clipped(0.8)	Clipped(0.8)	Clipped(0.8)

**Antipsychotics**					
	hERG	5-HT2A	5-HT2B	DRD2	CNS(0.5)
	Gaussian(0, 1.0)	Clipped(0.8)	Clipped(0.8)	Clipped(0.8)	—

The raw scores obtained from similarity or property measurements are post-processed by modifier functions that map the scores to the [0, 1] interval and allow the objective to be fine-tuned. The modifier functions used in this paper are *Clipped(value)*, *Gaussian(mean, variance)*, *MinGaussian(mean, variance)*, and *MaxGaussian(mean, variance)*. The *Clipped* modifier is a thresholded modifier in which values above a given threshold are mapped to one, while values below threshold decrease linearly to zero. The *Gaussian* modifiers target a specific value, returning high scores when the underlying value is near the target. The *Min* and *Max* versions of this modifier map the input value to one if it is lower or higher than the target value, respectively. For example, in the fexofenadine benchmark a molecule with a Tanimoto similarity higher than 0.8, a TPSA above 90.0 and a log(*P*) below 4.0 would score perfectly on each objective. More information on the modifiers can be found in the ESI† accompanying the Guacamol paper.^13^

Precise evaluation of generative models in terms of their value to pharmaceutical drug design programs can be challenging. To increase relevance, with respect to real-life drug design projects, while maintaining the efficient benchmark evaluations necessary for iterative design and statistical analysis, we integrate an existing data-driven surrogate model for target activity into the Guacamol benchmarking suite.^13^ We make use of a previously proposed surrogate model,^59^ minding the separation of concerns,^60^ that has been used to study failure modes in molecule generation. This model ranks molecules based on the ratio of trees in a random forest classifier, trained on ChEMBL activity data,^35^ predicting that the molecule is active. In the model, binary ECFP fingerprints^57^ of size 1024 and radius 2 are used as features.

In this paper, we provide two novel benchmarks for Pareto optimisation making use of this model. Inspired by the demands of a multi-target drug discovery project,^61^ we have constructed a multi-kinase inhibitor task and a multi-neuroreceptor binding antipsychotics task. In the kinase inhibitor task, we aim for molecules that inhibit three DAP kinases^62^ (DAPk1, DRP1, and ZIPk) often implicated in cancer while trying to avoid activity against common off-target ion channels^63,64^ (hERG, and SCN2A). In the ongoing search for novel anti-psychotic medication, focus has shifted^65^ to combined binders of serotonergic receptors (5-HT2A, and 5-HT2B) and a more classical target: the dopaminergic DRD2 receptor. In the multi-receptor antipsychotica task, we target these three receptors, and aim to avoid an off-target ion channel (hERG) while fulfilling the Pfizer central nervous system desirability requirements.

### 3.1 Dominated hypervolume

In multi-objective problems, tracking the evolution of an algorithm or measuring the quality of a Pareto front with respect to a single parameter can be challenging. In previous benchmarking efforts for optimisation algorithms of small molecules, the geometric mean of the objectives has traditionally been used as both an aggregate objective and as a metric. From a technical point of view, the geometric mean is the exponential of the arithmetic mean of the log-transformed set of objective scores. As a consequence, the geometric mean for strictly positive values is sensitive to severe underperformance in any single objective, making it a relevant measure for many multi-objective optimisation problems. However, other indicators of the quality of Pareto fronts have been developed by the multi-objective optimisation community. One such metric is the dominated hypervolume,^66^ which we introduce to the domain of chemical optimisation as an alternative measure for multi-objective optimisation benchmarks.

The dominated hypervolume (also known as Lebesgue measure^67^ or S-metric^68^) maps a set of points in objective space to the size of the region Pareto dominated by that set. The hypervolume has to be bounded from below by a reference point, which for the purposes of this paper will systematically be chosen to be the origin of objective space. The dominated hypervolume simultaneously takes into account the proximity of the points to the ideal Pareto front and their spread over the objective space. For problems with less than five objectives, the dominated hypervolume can be calculated exactly. However, for higher-dimensional multi-objective optimisation problems, calculating the dominated hypervolume precisely can be computationally expensive and hence a smorgasbord of efficient approximation methods^69,70^ for the dominated hypervolume has been developed.

### 3.2 Internal similarity

In comparing the performance of the different algorithms discussed in this paper, it is useful to differentiate whether algorithms encourage a significantly different amount of chemical diversity in their evolutionary populations. In cheminformatics, similarity between two molecules is usually quantified based on metrics applied to binary fingerprints that featurise chemical substructures. To calculate the diversity of molecules, the pairwise similarity of each combination of molecules in a set has been traditionally calculated using a binary similarity index, like the Tanimoto similarity,^54,55^ and summarised in an aggregate metric. However, the recent development of extended similarity metrics^71,72^ enables the simultaneous and straightforward comparison of an arbitrary number of bitvectors such as molecular fingerprints.

In this paper we make use of extended similarity indices to calculate and track the internal similarity of evolutionary populations. Extended similarity metrics, which compare a stack of bitvectors, have the advantage^71^ that they do not require the full similarity matrix of the compound pool or aggregate metric. In addition to being more efficient, extended similarity metrics reduce to the traditional binary similarity metrics if applied to a set of two molecules. According to computational experiments, two newly proposed extended similarity metrics^72^ are highly advantageous compared to the extended Tanimoto similarity: the extended Baroni–Urbani–Buser similarity index and the extended faith similarity index. Throughout this paper will make use of the extended faith similarity index.

## 4 Results

To increase the real-life relevance of the benchmarks used here, we run each algorithm 20 times for 150 generations per benchmark. We also reject molecules that either trigger the structural alerts from GSK,^73^ or those that contain ring allenes, macrocycles, an abundance of hologenicity (#F > 6, #Br > 3, #Cl > 3), rotatable bonds (>10) or hydrogen acceptors/donors (>10). In addition, the initial populations used in this paper consist of a hundred molecules randomly sampled from the Guacamol^13^ subset of ChEMBL.^35^ All these molecules are neutral, do not contain salts and have Tanimoto similarities below 0.323 to any of ten FDA approved drugs (celecoxib, aripiprazole, cobimetinib, osimertinib, troglitazone, ranolazine, thiothixene, albuterol, fexofenadine, mestranol).

Based on previous work comparing single objective optimisation methods, we choose GB-EPI (with geometric mean as surrogate fitness function) as a representative baseline to compare against NSGA-II and NSGA-III. For GB-EPI, we choose four medicinally relevant features of interest to span the archive: molecular weight (ranged from 140 to 555), log(*P*) (0.0 to 7.0), TPSA (0 to 140), and molar refractivity (40 to 130). For fair comparison, molecules exceeding these ranges are excluded from the evolutionary populations of NSGA-II and NSGA-III during the benchmarks. Based on previous experience with GB-EPI, the archive size for was set to 150 and the batch size to 20. The archive size in quality-diversity algorithms, such as GB-EPI, is the counterpart of the population size in traditional genetic algorithms. In general, the batch size refers to the amount of molecules submitted to mutation and crossover per generation. For NSGA-II, we used a population size of 100 (corresponding to the initial population) and a batch size of 20. For NSGA-III, we used the same batch size but experimentation guided us towards a smaller total evolutionary population: we settled on the use 25 reference directions, and a population size of 35 molecules. These hyperparameters were chosen to support global performance of each individual algorithm without disrupting splitting procedures, as a consequence the amount of fitness calls varies across algorithms and generations.

**Fig. 5:**
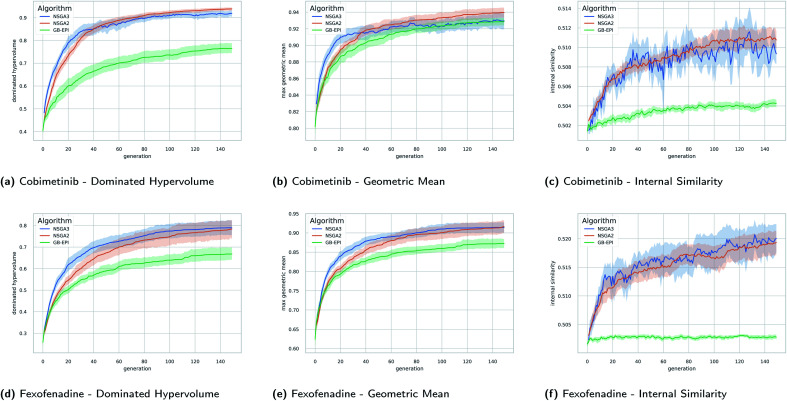
Timeseries plots with variance bands of the dominated hypervolume, the maximum geometric mean, and internal similarity for the cobimetinib (a–c) and fexofenadine (d–f) tasks as a function of generations of the evolutionary populations. The mean value (solid line) and the 95% confidence interval (variance bands) over twenty runs of NSGA-II (orange), NSGA-III (blue), and GB-EPI (green, optimising the geometric mean) are shown. Details of the experimental setup for these results, including hyperparameters, initial population and chemical filters are discussed in Subsection 4.

In Fig. 5 the evolution of the dominated hypervolume, maximum geometric mean and internal similarity of the NSGA-II, NSGA-III, and GB-EPI algorithms is shown for two representative benchmarks (cobimentib and fexofenadine). Throughout the computational experiments GB-EPI, which optimises directly for the geometric mean, is used as a baseline comparison method. As expected, NSGA-II and NSGA-III successfully out-compete the GB-EPI baseline in terms of dominated hypervolume for both benchmarks. In contrast to GB-EPI, the NSGA algorithms are designed specifically to optimise the Pareto front, the quality of which is measured by the dominated hypervolume. The geometric mean follows trends similar to the dominated hypervolume in the benchmarks. However, the values of the maximal geometric mean lie close to each other and the 95% confidence interval of GB-EPI overlaps with NSGA-II and NSGA-III during the latter stages of the cobimentib task.

An overview of the results for the multi-objective benchmarks is shown in Table 2 in terms of averages and standard deviations. NSGA-II and NSGA-III perform better than the baseline on each of the benchmarks for both dominated hypervolume and maximum geometric mean with the exception of the antipsychotics task. In that task, similarity between the three receptor targets disadvantages NSGA-III due to its rigid reference directions. For the fexofenadine and pioglitazone benchmarks, GB-EPI lies within one standard deviation of either NSGA-II or NSGA-III for both metrics. Note that to obtain the global maximum geometric mean of these benchmarks or the global optimum of one of the objectives, direct optimisation should be used. In principle, Pareto optimisation algorithms should reach these types of global optima, but significantly less efficiently as the evolutionary population is spread out over objective space. Conversely, when using a single aggregation function, the solutions tend to lie close to each other in objective space, and don't cover the entirety of the Pareto front.

**Table 2:** The dominated hypervolume, maximum geometric mean, internal similarity, and cumulative fitness calls after 150 generations, for seven multi-objective optimisation tasks averaged over 20 runs of the GB-EPI, NSGA-II, and NSGA-III algorithms. Details of the experimental setup for these results, including hyperparameters, construction of the initial population, and chemical filters are discussed in Subsection 4. Mean average values for each of the measures are given with standard deviations

Algorithm	Task	Dominated hypervolume	Geometric mean	Internal similarity	Fitness calls (cumulative)
**GB-EPI**					
	Cobimetinib	0.77 ± 0.05	0.93 ± 0.01	0.50 ± 0.00	13 577 ± 1224
	Fexofenadine	0.67 ± 0.07	0.87 ± 0.03	0.50 ± 0.00	17 985 ± 1398
	Osimertinib	0.54 ± 0.04	0.85 ± 0.01	0.50 ± 0.00	12 982 ± 1351
	Pioglitazone	0.98 ± 0.04	0.99 ± 0.01	0.50 ± 0.00	13 160 ± 3104
	Ranolazine	0.46 ± 0.04	0.81 ± 0.02	0.50 ± 0.00	16 859 ± 1537
	DAP kinases	0.03 ± 0.05	0.46 ± 0.06	0.51 ± 0.00	23 545 ± 3150
	Antipsychotics	0.09 ± 0.02	0.57 ± 0.06	0.51 ± 0.00	21 905 ± 3073

**NSGA-II**					
	Cobimetinib	0.94 ± 0.02	0.94 ± 0.01	0.51 ± 0.00	17 784 ± 1753
	Fexofenadine	0.78 ± 0.10	0.92 ± 0.04	0.52 ± 0.00	20 268 ± 2909
	Osimertinib	0.66 ± 0.03	0.89 ± 0.01	0.52 ± 0.00	16 848 ± 2655
	Pioglitazone	1.00 ± 0.00	1.00 ± 0.00	0.51 ± 0.00	19 944 ± 4765
	Ranolazine	0.68 ± 0.06	0.87 ± 0.02	0.51 ± 0.00	21 259 ± 2181
	DAP kinases	0.05 ± 0.03	0.50 ± 0.07	0.52 ± 0.00	24 350 ± 3826
	Antipsychotics	0.08 ± 0.03	0.50 ± 0.05	0.51 ± 0.00	21 246 ± 1909

**NSGA-III**					
	Cobimetinib	0.92 ± 0.03	0.93 ± 0.02	0.51 ± 0.00	14 224 ± 1807
	Fexofenadine	0.79 ± 0.00	0.91 ± 0.03	0.52 ± 0.01	12 950 ± 2326
	Osimertinib	0.66 ± 0.03	0.89 ± 0.01	0.52 ± 0.00	11 052 ± 2337
	Pioglitazone	1.00 ± 0.00	1.00 ± 0.00	0.51 ± 0.01	10 639 ± 2736
	Ranolazine	0.63 ± 0.06	0.85 ± 0.02	0.51 ± 0.00	17 949 ± 2732
	DAP kinases	0.04 ± 0.02	0.48 ± 0.07	0.51 ± 0.01	22 454 ± 3440
	Antipsychotics	0.05 ± 0.03	0.49 ± 0.04	0.52 ± 0.01	32 991 ± 3473

To study the comparative efficiency of each algorithm, we track the cumulative number of function calls over the full 150 generations for the twenty individual runs of each algorithm. This has the advantage that it does not interrupt the splitting front procedure, as might be the case when working with a fixed and limited function call budget. An overview of the mean and standard deviation of the cumulative fitness calls of each algorithm is shown in Table 2. NSGA-III consistently outperforms NSGA-II in terms of efficiency, and is more efficient than GB-EPI in all benchmarks where they have similar performance for dominated hypervolume and geometric mean. In contrast to single objective optimisation problems, where a lower internal similarity has been regarded as beneficial, for multi-objective optimisation the algorithms which encourage greater internal similarity are better performing.

## 5 Conclusion and outlook

This paper introduces two novel open-source and graph-based implementations of non-dominated sorting genetic algorithms, NSGA-II and NSGA-III, for small molecule multi-objective optimisation. The performance of these algorithms is compared to a single objective quality-diversity algorithm (GB-EPI) on four metrics: dominated hypervolume, maximal geometric mean, internal similarity and efficiency. Previous benchmarks for generative models of small molecules focused on the maximal geometric mean as a sole aggregate indicator of success in multi-objective optimisation. However, the Pareto front – the collection of optimal points in objective space – is not solely characterised by the geometric mean of a single molecule. In this paper we show that the size of the hypervolume dominated in objective space (with respect to the origin) is a useful, often more discriminative, alternative metric in generative model benchmarks.

The performance of NSGA-II and NSGA-III for graph-based optimisation of molecules is encouraging. Both algorithms specialise in finding the optimal Pareto front and our benchmarks show that this approach is superior compared to GB-EPI (which optimises the geometric mean directly). In line with analyses of purely numerical benchmarks found in the literature, NSGA-III does not always outperform NSGA-II in our chemical benchmarks, indicating that the two algorithm produce similar results according to this metric. Throughout all the benchmarks presented in this paper however, NSGA-III seems to be the most efficient in its use of function calls. Notably, and in contrast to single objective optimisation, the higher performing algorithms NSGA-II and NSGA-III have a higher and faster increasing internal similarity in their evolutionary populations than the baseline.

The above discussed efficiency, performance, and flexibility of the graph-based implementations of NSGA-II and NSGA-III for small molecule multi-objective optimisation as provided with this paper, allows the community to use these algorithms for practical use. In addition, these implementations can be used as future baselines and as starting points for future developments in this field. One such possible development would be to further reduce the amount of function calls through the use of contextual multi-armed bandits,^74^ or Gaussian processes^75^ to prune the amount of molecules presented to the evaluation step of the algorithms. Finally, the algorithms presented here can be integrated into the workflow for multi-objective tasks given to self-driving laboratories^76^ or other set-ups making use of active learning.^77^

## Data availability

Full code for the implementations of NSGA-II and NAGA-III is available at: https://github.com/Jonas-Verhellen/MolecularGraphPareto.

## Author contributions

The author confirms sole responsibility for the following: study conception and design, data collection, analysis and interpretation of results, and manuscript preparation.

## Conflicts of interest

There are no conflicts to declare.

## Supplementary Material

SC-013-D2SC00821A-s001

SC-013-D2SC00821A-s002
